# Single-Crystal Optical Fiber-Based Raman-Distributed Temperature Sensing for Applications in Energy Systems

**DOI:** 10.3390/s26216786

**Published:** 2026-10-27

**Authors:** Michael Buric, Gary Lander, Dolendra Karki

**Affiliations:** 1 National Energy Technology Laboratory (NETL), United States Department of Energy, Morgantown, WV 26505, USA; glander1724@gmail.com (G.L.); dolendra.karki@netl.doe.gov (D.K.); 2 National Energy Technology Laboratory (NETL) Research Support Contractor, Morgantown, WV 26505, USA

**Keywords:** single crystal optical fiber, laser heated pedestal growth, crystal growth, dopant segregation, optical cladding, Raman distributed temperature sensing, high-temperature sensors

## Abstract

Modern energy generation systems often exhibit some of the most challenging measurement environments on Earth. Coal, oil and gas, or nuclear based systems can present extremely high-temperatures, corrosive or erosive environments, extreme radioactivity, a wide-range of pressures, and extreme levels of vibration. To access the highest value components for sensor applications in these systems, we implement a distributed fiber optic temperature sensing system that measures temperature variations along the full length of the sensor fiber. In contrast to standard distributed fiber sensors, this system uses single-crystal optical fibers made from sapphire or yttrium aluminum garnet as the sensor element. Here, we provide a perspective and overview of our team’s combined efforts to grow single-crystal sapphire optical fibers with 125 µm outer diameters and a range of core sizes via the laser-heated pedestal growth (LHPG) method and refine or clad those fibers using a second LHPG system. These efforts are integrated with our Optical Interrogator Development Program, in which we construct distributed fiber-sensing interrogator units that are tailored to specific energy applications. In closing, we detail recent comparisons with our Raman distributed temperature sensing system and commercially available distributed sensing equipment.

## 1. Introduction

Modern coal, oil and gas, and nuclear based generation systems continue to achieve higher operating temperature regimes. This trend has largely been driven by the intrinsic desire to increase the Carnot efficiency of generation systems, combined with achievements in materials science and cooling system technologies. Coal-fired boiler systems operating in the 1960s were largely subcritical systems, which exhibited steam temperatures approaching 600 °C, with efficiencies near 30%. The advent of super-critical and ultra-super-critical steam systems saw increases past the 600 °C mark, with efficiencies moving beyond 40% [1,2]. Increases were made possible by advances in nickel super-alloys used in boiler tube systems and other boiler components [3]. Gas turbine-based systems saw increases in combustor operating temperatures from F-class systems in the 90′s operating at 1200–1400 °C, to modern H or J class systems firing between 1500 °C and 1650 °C. These advances were also driven by nickel-based super alloys, as well as the advent of single-crystal turbine blades. Further advances in thermal barrier coatings and blade cooling systems helped push the operating temperatures, as well as the thermal efficiencies, higher over time. Although the majority of nuclear reactors are currently light-water reactor designs, advanced nuclear reactor designs are following a similar trend. While light water reactors generally operate near 300 °C, sodium-cooled reactor designs may operate somewhere above 500 °C, and lead-cooled designs may operate up to about 800 °C. Molten salt-based reactor designs will likely see outlet temperatures approaching 850 °C. Coal fired boilers, gas turbines, and nuclear reactors account for more than 75% of the energy generated currently in the United States [4]. Given trends in all three of these key energy generation categories, it is highly likely that power generation systems will continue to push the boundaries of high-temperature operation and will require new and innovative methods to maintain safe operation at these extremely high-temperatures.

The operations of these advanced energy systems are governed, controlled, and monitored by a vast array of sensing and control systems. Safe and efficient operation of ultrahigh-temperature systems requires careful monitoring of temperatures, pressures, fuel utilization, and mechanical operating parameters. A few key areas in sensor technology have emerged to tackle the extreme environments presented by these harsh environments. Thermocouples are widely used, with S and B types being most common. They are capable of measuring temperatures up to about 1650 °C but are often noted as being temporary devices that require frequent or periodic replacement due to junction degradation. Advanced nickel alloys also play a significant role in providing packaging solutions for various types of high-temperature sensors but also face temperature limitations and longevity considerations. Advanced ceramics are also often employed as packaging solutions, although thermal mismatches and susceptibility to vibration can limit deployment. The emergence of wide-bandgap, high-temperature semiconductors has enabled some systems to employ semiconductor-based sensing up to about 1000 °C, but challenges with placement, vibration, and interconnection wiring or communication present significant limitations [5]. Finally, advances in remote or standoff detection, such as thermal imaging, have enabled some devices to operate outside the hot zone while maintaining a view of internal temperatures or other parameters. The highest-value data are obtained through the most direct measurement, which means accessing the most challenging harsh environments directly. This fundamental principle of sensors and controls dictates that we attempt to measure the highest temperatures closest to the core of the thermal system. The second fundamental truth dictates that we take enough measurements at a fast enough rate to characterize the intrinsic complexity and dynamics of a given system. This implies that the spatial and temporal resolution of a sensor system match both the minimum physical dimension of any variation in the measurement zone, as well as the frequency of any transient that can be generated. The spatial requirement is made more challenging as energy generation systems span from ever smaller micro-reactors to even larger capacity mega-plants. The temporal requirement is made more challenging with new types of control systems that operate with greater speed and precision. These concepts drive our continued research and development into the application of new types of harsh-environment fiber optic sensors in advanced energy generation systems.

In this paper, we will detail some of our ongoing and recent efforts aimed at expanding the capabilities of fiber optic sensors into the harsh environment realm of energy generation systems. To achieve this end, we have initiated a robust program in single-crystal optical fiber distributed sensing. Researchers have long appreciated the robust high-temperature capabilities of crystalline materials. These single-crystal materials are capable of extreme temperature operation, have resistance to chemical erosion, and can be resistant to some types of radioactivity [6,7,8,9]. We can see a direct parallel between the introduction of single-crystal turbine blades to increase turbine engine operating temperatures and the introduction of single-crystal optical fibers to increase sensor temperature measurement capabilities. Two of the primary candidates for single-crystal fiber implementation are sapphire and yttrium aluminum garnet (YAG). Both materials exhibit high-melting temperatures at about 2040 °C and 1970 °C respectively. These values are high enough to merit consideration in the most challenging high-temperature environments. Sapphire is arranged in a hexagonal crystal structure, while YAG presents a cubic crystal lattice. Given those crystallographic structures, YAG is isotropic or has similar properties regardless of orientation, while sapphire is anisotropic and presents optical properties that are different for each crystal axis. These two materials present a wide range of opportunities for sensor and fiber configurations for harsh environments [10,11,12]. They are the two forms of crystal most commonly applied to applications with extreme power and heat. Sapphire has been used extensively as window material for high-temperature systems, while YAG is most commonly used as a laser crystal due in part to its high-thermal conductivity enabling effective cooling of the YAG laser gain medium. This reasoning plays a major role in our exploration of these two crystal systems for high-temperature sensors.

## 2. The LHPG Method for Single-Crystal Fiber Production

**Figure 1:**
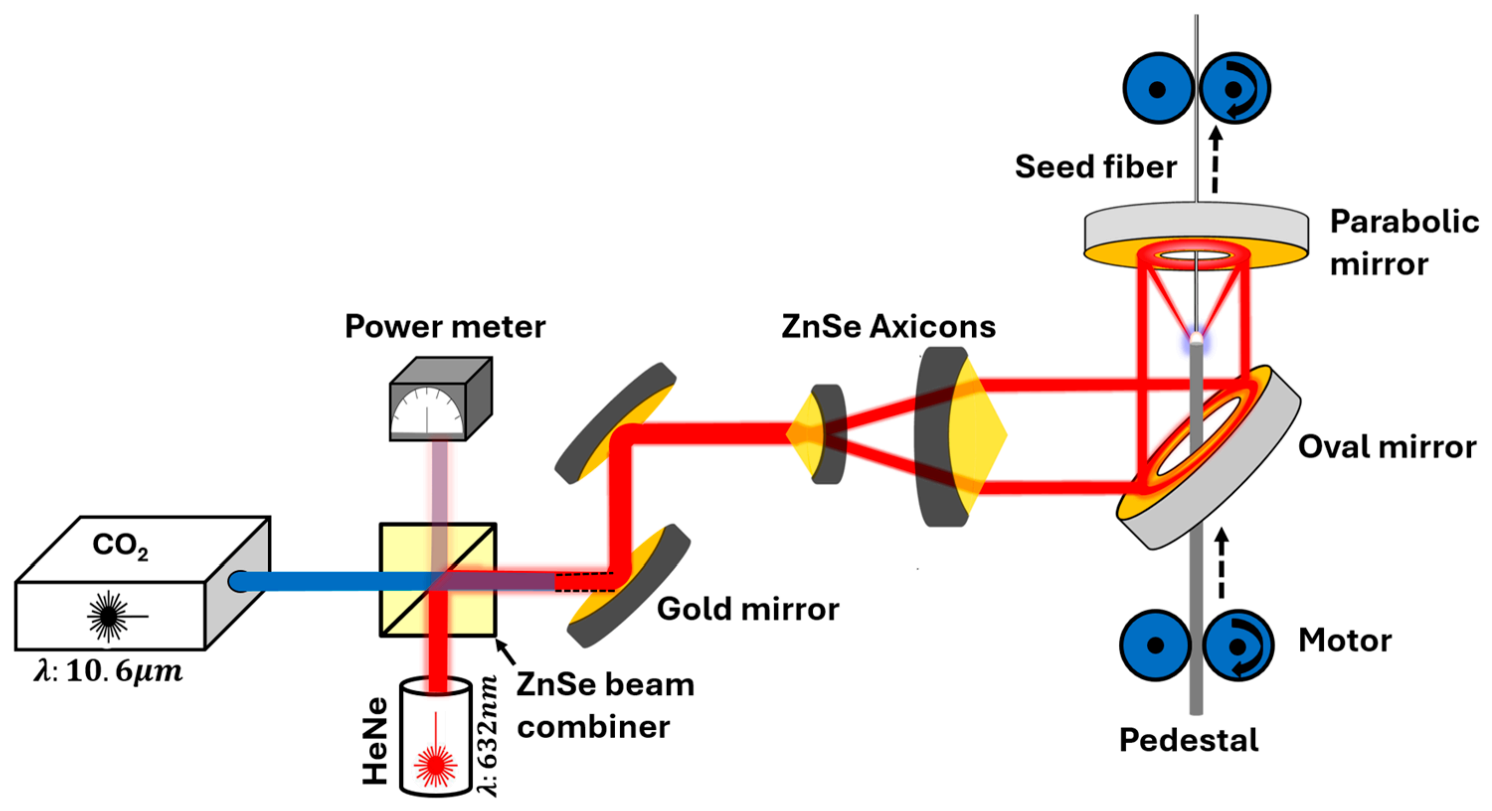
Schematic of the laser-heated pedestal Growth system. A CO_2_ laser is used as a heat source to melt single crystal material from which the fibers are grown.

The gold standard to produce single crystal fibers has long been the laser-heated pedestal Growth (LHPG) method [13,14]. A typical LHPG system is depicted in the schematic in Figure 1.

In this method, the end of a precursor rod or preform, known as a “pedestal” is melted by a high-power laser system. CO_2_ lasers are often employed as the heat source for such systems because of their stability, high continuous-wave power, and high optical absorption by both sapphire and YAG at the 10.6 µm CO_2_ laser line. Indeed, both materials are so absorptive at this wavelength that most of the incident 10.6 µm light is absorbed within 1 µm of an incident surface. A modern LHPG system includes a set of optics designed to uniformly distribute laser light onto the tip of a pedestal rod to enable even melting of the preform as well as a uniform thermal gradient in all directions. The most common means of achieving such distributions is using a pair of transmissive axicons or reflaxicons (reflective axicons). These are cone-shaped optical elements. Axicon materials need to be transparent to the 10.6 µm line and are therefore often made of zinc selenide (ZnSe), while the reflaxicon version must be reflective to the laser line and are therefore often coated with gold. In LHPG, the first incident axicon transforms a regular Gaussian CO_2_ laser beam into an expanding doughnut shape or annular beam. That expanding beam is then passed through a second axicon element with cone angles that are the same as the first axicon. The result is a collimated ring or annular beam. The purpose of such elements is simply to provide a laser beam structure which is axially symmetric and provides a convenient “through hole” in the middle which allows physical contact between the pedestal and the seed fiber. A standard parabolic mirror will focus this annular ring into a smaller annular ring, which creates a convenient location at which the single crystal fiber being grown will emerge without being re-melted by the laser heating the pedestal. Since terrestrial LHPG systems are always affected by gravity, growing uniform small-diameter crystals fiber requires growth in the vertical orientation. This leads most LHPG system designers to employ a flat or “turning” mirror at a 45° angle of incidence to turn a horizontally propagating annular beam into a vertical beam; acting on a vertically oriented pedestal. The parabolic focusing mirror is often placed on top of the pedestal material and redirects CO_2_ laser light traveling in the upward direction into a beam focusing downward. During growth, the pedestal tip melts, and a small seed crystal is lowered into the molten zone. In the first known system, this seed crystal was a small fragment of a larger bulk crystal. In modern variations, the seed crystal is often just a short length of previously grown fiber of the same material and desired crystalline orientation. While the optical systems for LHPG are highly specialized, they are not overly complicated. The remainder of the components needed for high-quality LHPG constitute the mechanical drive and fiber measurement systems.

**Figure 2:**
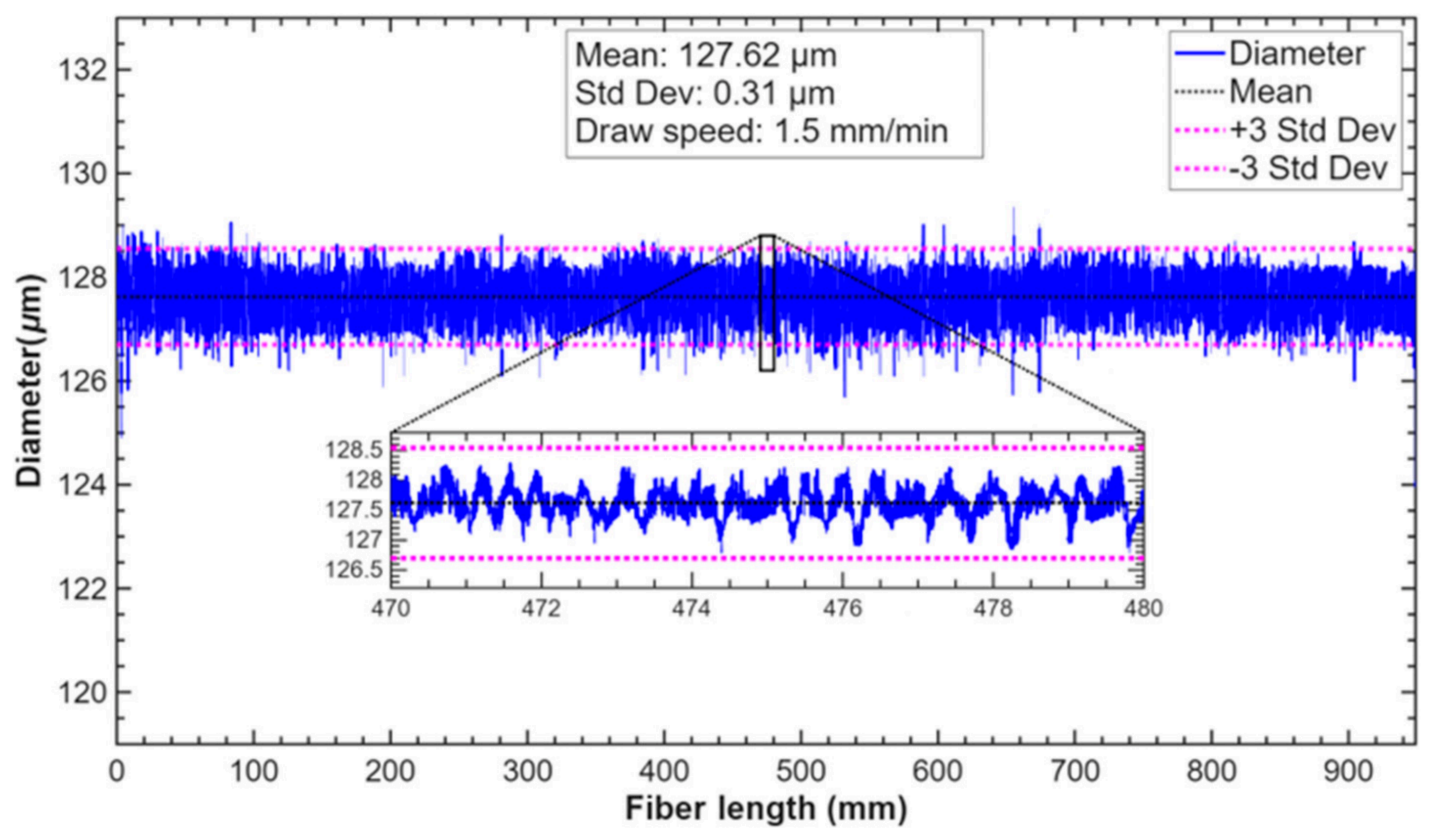
Diameter variation in a one-meter long ~127 µm-diameter sapphire fiber grown via the LHPG method, with inset providing a magnified view over a shorter section.

To produce single-crystal optical fiber with good optical transmission and mechanical properties using LHPG, the system must include high-precision motion control and fiber measurement systems. During growth, it is necessary to move the pedestal material upward to replenish the portion continuously melted and consumed into grown single crystal fibers. The fiber must also be pulled upwards out of the molten zone. Due to the conservation of mass in the system, the proportion of the velocities of the pedestal and fiber materials are approximately equal to the proportion of the cross-sectional areas of the pedestal and fiber. Diameter reductions of about 3:1 is common due to the intrinsic stability of the liquid meniscus at that ratio. This implies a 9:1 ratio in the velocity of the grown fiber to the pedestal velocity. It also implies a 1:9 ratio of the length of pedestal consumed to the length of fiber produced. The speed at which LHPG can produce fiber is in the order of millimeters per minute, depending on a range of factors including ambient atmosphere, grown fiber diameter, etc. This is a major factor driving the cost of single-crystal fiber sensors and devices. The speeds generated by motion components are therefore relatively low and require high precision. The systems we have constructed explicitly utilize brushless, slot-less, DC electric drive motors with optical encoders and closed-loop control for the pedestal and fiber speeds along with harmonic gear reducers operating at a 100:1 ratio. This permits smooth continuous drawing at the slow speeds that is key to the production of high-quality fibers. The second important design factor is the accurate measurement of micron-scale fibers in diameter during the growth process. Because small variations in factors such as laser power, pedestal diameter, or even ambient temperature can affect the diameter of the finished fiber, it is necessary to carefully measure the diameter of the fiber as it exits the molten pool and adjust one or more factors to maintain fiber diameter along a long length of fiber that may take hours or tens of hours to produce. We have found that the best control method for LHPG involves careful control of the laser power, pedestal translation rate, and grown fiber translation rate [15,16]. These three factors tend to affect finished fiber diameter in that same order; decreasing in magnitude of effect. In other words, laser power can have the most dramatic influence on the fiber diameter, so it is kept as constant as possible with active closed loop control. The pedestal translation rate is the second-most influential parameter in terms of its effect on fiber diameter, and it is actively used to suppress system perturbations by continuously adjusting its velocity through a proportional-integral-derivative feedback loop to maintain a uniform diameter at a fixed upward fiber-draw speed. This motion control system provides an accurate means to produce single-crystal optical fibers with less than 1% variation in diameter (see Figure 2, showing diameter variation within 3 standard deviations (or around 1% of diameter) in newly grown sapphire fiber of mean diameter 127.62 µm).

It has also permitted us to produce 20 m long fibers. While fibers longer than 20 m are practically feasible [17], the useful length is limited by the optical losses in the crystalline material, which means longer fibers may not be useful due to low transmitted signal. Future advances in loss mitigation may enable fiber lengths exceeding 20 m to become practical.

**Figure 3:**
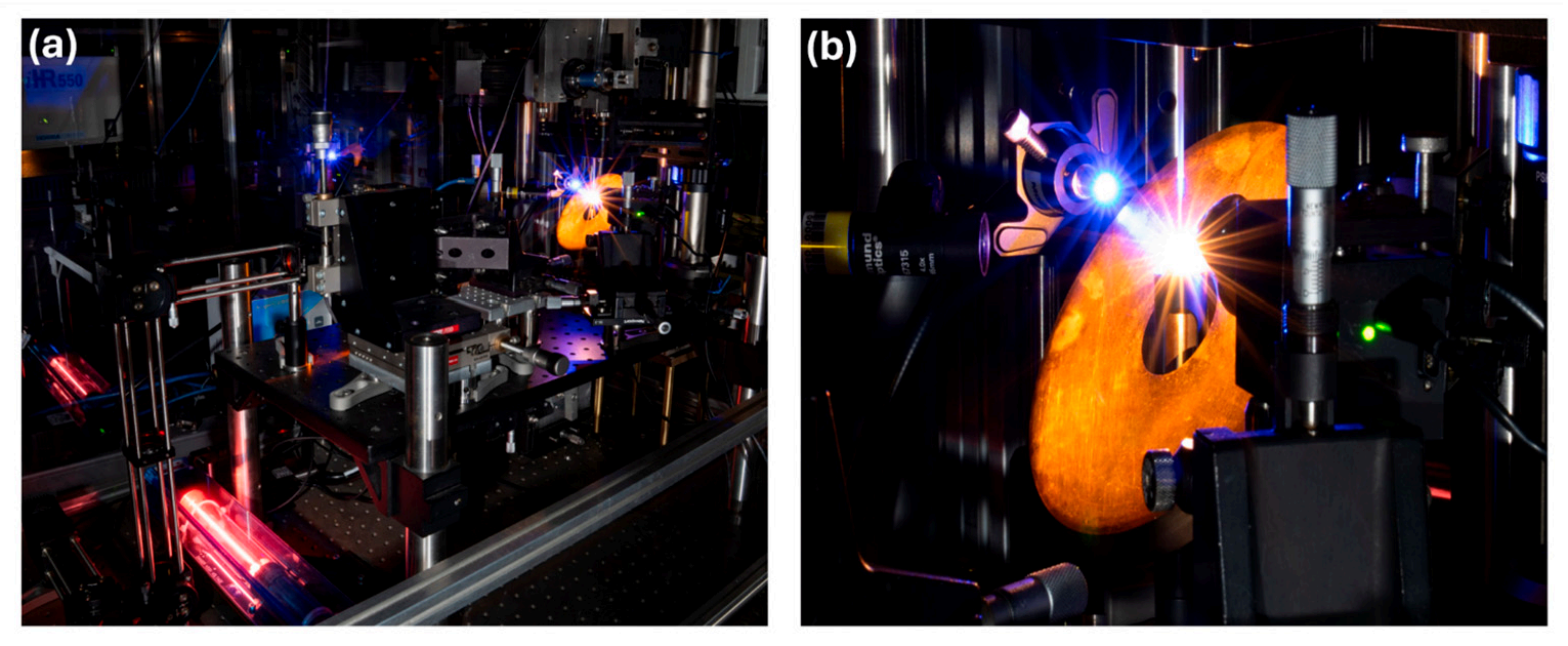
LHPG system photos. (**a**) System-wide view and (**b**) Close-up of molten zone and turning mirror.

The final component in the LHPG system required to produce high-quality single-crystal optical fibers is an accurate fiber diameter measurement system. Our preferred method is the use of machine-vision cameras focused on the molten zone. These cameras operate at a large standoff distance such that the camera lens is outside the CO_2_ beam and does not inadvertently absorb the edges of the annular beam, which is roughly 8 or 9 cm in diameter in the collimated beam path. We also employ machine-vision telecentric lenses for accurate measurement of the finished fiber and molten zone. Telecentric lenses maintain constant image size and magnification while eliminating parallax errors enabling high-accuracy diameter measurements. This has the effect of placing more of the diameter of the fiber in focus, even though we are viewing the fiber from the side. Although the sapphire and YAG fibers are somewhat round in cross section, they often feature hexagonal or square corners due to their crystalline structure. In principle, a perfect unbroken crystal would be perfectly hexagonal or square, respectively. Use of telecentric lenses in measuring these shapes allows more precise measurement of the diameter of the finished fiber. Furthermore, we generally employ two such cameras and lenses positioned at 90 degrees to one another. This allows us to average out any difference in diameter due to the somewhat hexagonal or square structure being measured (Figure 3).

Our LHPG systems utilize machine vision techniques to accurately characterize measured diameters from image data. First, we employ a blue (463 nm) light emitting diode backlight system to illuminate the molten zone. This results in collected images in which the background appears light, and the fiber itself appears dark. A matched set of filters integrated with the camera lenses permits collection of the backscattered light, while eliminating the ever-present blackbody radiation from the over 2000 °C molten pool. Without this added lighting system, it is more difficult for machine vision algorithms to delineate the background from the grown fiber. The machine vision algorithm relies mostly on edge detection to find the edges of the fiber, and to measure the grown fiber diameter. Because backlit fiber exhibits high contrast, even though it is very hot and visibly glows (similar in appearance to an incandescent light bulb filament), accurate measurement is possible. Once the grown fiber edges are found, the fiber diameter is readily calculated. The measured diameter is then used as feedback control to adjust pedestal speed to minimize the diameter variations. An inverse relationship exists, i.e., when the grown-fiber diameter falls below the target value, the feedback loop increases the pedestal velocity, whereas an oversized diameter triggers a corresponding reduction in pedestal speed. This two-camera measurement system is robust and can be programmed with features such as automatic error correction in which improperly grown sections of fiber are re-melted and grown again, or with stop/start functionality which permits stopping a long fiber growth to change pedestal material once one pedestal is exhausted.

## 3. Optical Cladding on Single Crystal Fiber via the LHPG Method

Standard step-index optical fibers are axially symmetric devices with two distinct indices of refraction. Fiber cores present a slightly higher optical index, while the index of refraction in the cladding is slightly lower. This well-known structure permits the efficient waveguiding and propagation of light inside a fiber for long distances. A single-crystal optical fiber does not intrinsically possess this property unless modified or altered. Unaltered single-crystal fibers are often referred to as “un-clad” “air-clad” or “no-core”. A sapphire crystal itself exhibits a high refractive index (RI) of about 1.77 at 532 nm parallel to the c-axis. The air surrounding a sapphire crystal fiber (RI slightly greater than 1 at the same wavelength) therefore acts as a cladding and confines light in the fiber. This allows crystal fibers to propagate light in free air, as long as the fiber does not come into contact with dirt, contaminants, or materials with higher indices of refraction. This is of course non-ideal for harsh-environment sensor applications, because all the target energy systems we discussed previously contain a plethora of these contaminants, and maintaining a clean fiber system with only air-cladding requires careful packaging. That method precludes the use of any bare fiber surface where the goal is to measure chemistry or requires direct contact with the fiber surface. This is why a major problem in single-crystal fibers has become the application of claddings. Claddings will ideally exhibit an optical index of refraction slightly lower than that of the crystalline core material, while maintaining similar robustness, temperature handling capabilities, and physical properties. Cladding the fiber also drives the reflection boundary from the surface to inside the crystal, where surface roughness no longer affects the propagating modes. Researchers have demonstrated a wide variety of methods for cladding single-crystal fibers including the application of various layers of materials including glasses, polycrystalline materials, metals, and others [18,19]. Although some of these techniques are highly promising and useful, none of them maintain all of the desired operational characteristics of the bare crystal without adding some limitations.

Our preferred method for the generation of clad single-crystal fiber is very similar to the method used for regular silica optical fibers. We rely on the use of dopants distributed within the crystal to alter the index of refraction and enable a core/cladding structure. In standard silica optical fibers, core dopants are used to increase the index of refraction, while cladding dopants are used to decrease the index of refraction. The same holds true for single-crystal optical fibers, although the method of dopant distribution is far different. Dopants can be applied either in the molten phase, or in the solid phase. Doping in the solid phase generally requires raising the temperature of the crystal to at least about two-thirds of its melting temperature. Dopants in that scenario can be applied to the surface as solids and permeate inside the material through the surface due to thermal diffusion. This is generally the method attempted at cladding-doping in crystalline fibers. An alternative method of cladding engineering is doping in the molten phase. This method requires that the host crystal material as well as the dopant are both added to the molten phase and are arranged into a core/cladding structure. This method is made possible by thermal convection current or “Marangoni current” combined with the solid/liquid segregation coefficient of the dopant in the solidifying molten zone of an LHPG system [20]. Because the LHPG system uses a focused beam of CO_2_ laser heat source that is applied to the edges or periphery of the molten zone, a strong thermal gradient exists from the edge to the center of that zone. This causes material to naturally be churned by the thermal gradient [21]. Various dopant materials react differently in the molten zone and tend to segregate with respect to the magnitude of their segregation coefficient at the solidification front. A lower segregation coefficient (K < 1) means more dopant ions will be present in the liquid than in the solid phase. The coefficient tends to be the lowest for larger ions with the same charge. In other words, dopant species with low segregation coefficients are pushed in greater numbers into the remaining liquid phase as solidification occurs. The periphery of the fiber solidifies first, and therefore contains less dopant ions than the core, which solidifies last upon cooling and contains the largest number of these dopant ions. This means that we can select an appropriate ionic species to add to the growth pedestal, and it will naturally concentrate towards the center of a grown fiber as it grows.

We have engineered a facility specifically to exploit this fiber doping technique and use it for the production and testing of single-crystal fiber formulations. Therein, we explore and optimize single-crystal fibers for a range of sensor applications. The goal is to produce fibers that exhibit low loss at certain wavelength regimes of interest, while maintaining robust high-temperature properties. To accomplish this, we constructed a unique tandem LHPG system. The first LHPG operates at a laser power of about 50 watts and enables the use of 1 mm diameter pedestal materials. These pedestals are grown into fibers approximately 300 µm in diameter. A 10-cm pedestal produces roughly 1-m of finished pure, single crystal fiber material. Solid phase materials are then applied to the outside surface of the 300 µm first-stage fiber, often via the sol-gel method [22]. Other applicable but less often used methods include physical vapor deposition, chemical vapor deposition, and liquid phase epitaxy methods. Once dopant materials have been applied to the outside of pure fibers, they are next re-grown using our second-stage LHPG system. In the second stage, 300 µm diameter fibers are used as pedestals. The laser system provides less than 10-watts of power, which is adequate for melting these already sub-millimeter scale fibers. Therein, dopants that were applied to the outside of the first-stage fiber are incorporated into the melt and driven by the molten-zone convection currents segregating the solidification front toward the center of the second stage finished fiber. These second-stage growths are generally used to produce fibers of diameters 125 µm or smaller. Variation in growth parameters can help create smaller modal volumes in the fiber, although achieving single-mode operation in the visible regime is still a highly challenging problem that has yet to be adequately demonstrated. The overall uniformity of crystalline fibers clad using this method is still an active topic of study in our research and will likely be reported extensively in the near future. While measurements of total attenuation and output mode structures is fairly straightforward, measurements of core sizes along the length requires destruction of the fibers and subsequent microanalysis. We note that a wide range of core sizes from approximately a third to 90% of the finished fiber diameter are possible. Optical losses also vary widely with dopant type and concentration. The fiber discussed in example experiments here achieved approximately 90% optical throughput including reflection and transmission losses. Once fibers are doped and re-grown via the second stage, they are ready for use in various types of single-crystal fiber sensors and harsh-environment applications.

## 4. Raman-Distributed Temperature Sensing for Extreme Environments

Researchers have invented numerous ways to utilize single-crystal fibers as sensor devices. Notable examples include single-crystal optical fiber temperature, pressure, chemical, displacement, and vibration sensing devices [23,24]. One of our long-term goals has been to introduce fully distributed fiber optic sensing to energy generation systems. To accomplish this lofty goal, we obviously could not rely on fused silica fibers due to their intrinsic low melting points and severe degradation of optical and material property due to dopant diffusion at moderate temperatures. The highest temperatures of long-term operation for those fused silica systems operated in air or in the presence of humidity are around 700–800 °C, while employing specialized metal or boron nitride coatings and ensuring that fibers are not stressed during operation. However, even unclad sapphire and YAG permit significantly better measurement capabilities and operational temperature limits. As we mentioned, these fibers are also generally multimode at most wavelength regions of interest. This feature drives the types of distributed interrogators we can apply to single-crystal distributed fiber sensors.

Distributed optical fiber sensor interrogators generally fall into a few primary categories including optical frequency domain reflectometry (OFDR) and optical time-domain reflectometry (OTDR). The frequency-domain based systems rely on interferometry and tunable lasers to calculate measurands at a specific length or distance down the fiber under test. This implies that OFDR systems rely on single-mode optical fibers. This fact basically rules out the use of OFDR for intrinsically multimode single-crystal fibers. OTDR, however, relies on pulsed lasers and time-of-flight measurements to determine measurands at a particular length down the fiber. This is primarily the reason behind preferring a Raman-distributed temperature sensor (Raman-DTS) system explicitly suited for single-crystal optical fibers. To our knowledge, There have been no reports in the literature of other types of fully-distributed sensor systems for use with single-crystal optical fibers. The specific novelty of combining single-crystal optical fiber with Raman-DTS is therefore to achieve higher temperature measurements than those that are possible using silica fibers.

**Figure 4:**
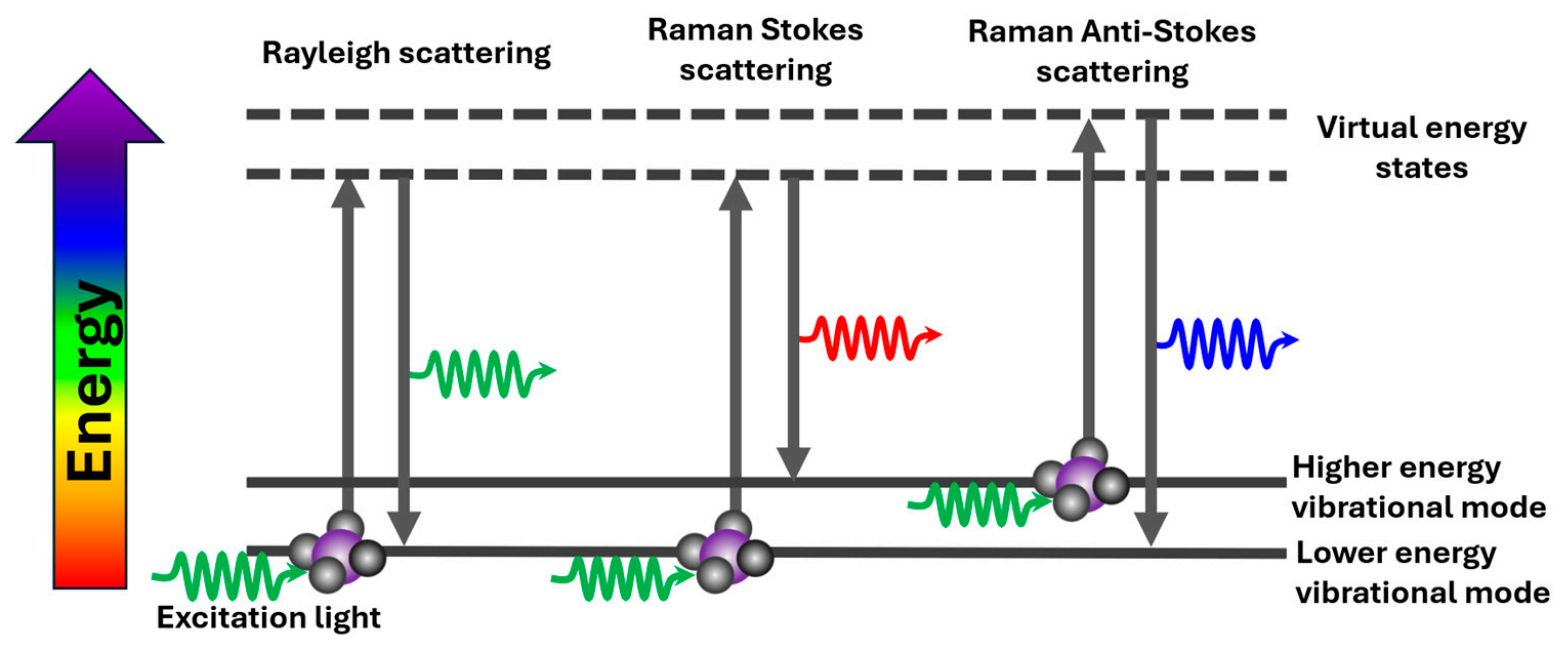
Energy level diagram depicting Rayleigh and Raman scattering. Raman scattering is an example of inelastic scattering in which the scattered photons have a different energy than the excitation photons. The colors signify that the Stokes radiation is red-shifted with respect to the excitation source while the anti-Stokes radiation is blue-shifted.

The first known DTS system for sapphire optical fiber was constructed at Virginia Tech [25] and used for basic measurements of temperature using short lengths of commercially available sapphire optical fiber from Micromaterials Inc. [26]. Bose-Einstein statistics govern the thermal population of vibrational states and thus set the temperature-dependent ratio of Stokes to anti-Stokes photons generated via Raman scattering. An energy level diagram depicting the difference between Rayleigh and Raman scattering is depicted in Figure 4.

If we measure the ratio of anti-Stokes to Stokes photons emitted from an exciting laser pulse, we can determine the temperature of the target material. Operation of our Raman-DTS system follows the same procedure where:

$$\frac{I_{AS}}{I_{s}}={\left(\frac{\nu_{L}+\nu_{R}}{\nu_{L}-\nu_{R}}\right)}^{4}e^{\frac{-h\nu_{R}}{kT}}$$

and *T* is the absolute temperature in Kelvin, $I_{AS}$ and $I_{s}$ are the anti-Stokes and Stokes line energies respectively, $\nu_{L}$ is the laser frequency, $\nu_{R}$ is the Raman shift frequency, h is Planck’s constant, and k is Boltzmann’s constant. The system is calibrated in one step by which the fiber is held at a constant temperature and a single coefficient is established at each point along the length to normalize the measured Stokes/anti-Stokes ratio, resulting in reporting of calibrated temperature thereafter. A laser pulse is focused into a single-crystal optical fiber. The pulse results in Raman scattering which travels in both directions down the fiber as it is generated. At the interrogator, dichroic beam splitters channel the Stokes and anti-Stokes light into separate avalanche photodiode detectors which are sampled at a GHz rate. Group velocity in the fiber is used to translate the time of flight into distance along the fiber under test. The temperature-dependent ratio of anti-Stokes to Stokes amplitude is measured in time domain and converted into temperature profile along the fiber [27,28,29]. Because this type of measurement relies on a ratio of Stokes and anti-Stokes amplitudes, varying attenuation along the fiber does not affect the measurement accuracy, as long at the attenuation is roughly the same for both spectral components. Because the components are relatively close together with the chosen pump wavelength in the green, we find that they do not attenuate at significantly different rates over the range of temperatures described here. This enables the use of a single calibration matrix coefficient applied to each recorded Stokes/anti-Stokes ratio point along the fiber length. Furthermore, the only normalization procedure used was to remove any DC bias from detected signals.

**Figure 5:**
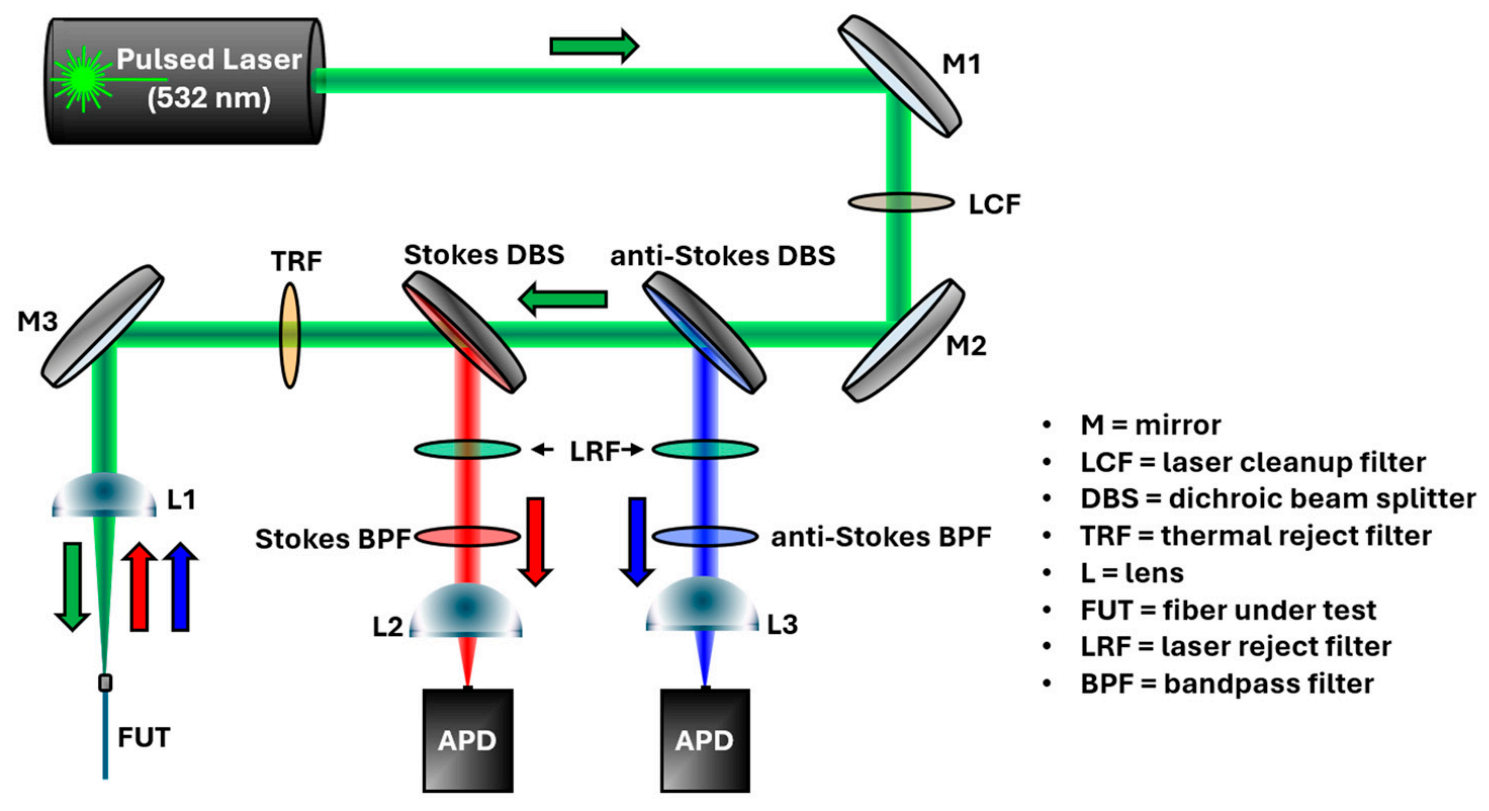
Schematic of custom-built Raman-DTS system. Green: pump laser beam path, Red: Stokes beam path, Blue: anti-Stokes beam path.

Our Raman-DTS system was designed to maximize utility for a range of energy generation systems and components. A schematic of the system is provided in Figure 5.

Therein, a 532 nm laser with ~350 ps pulse width and energy ~4.7 µJ per pulse and a peak power of ~13 kW is used as the excitation source. An aspheric achromat is used to couple the laser pulse into a single-crystal optical fiber. This lens must be optimized for use with the fiber under test. Because of the relatively low Raman cross sections of sapphire and YAG, high-energy laser pulses are desirable to maximize Raman scattering. However, high-power densities at the input facet of the fiber can damage the fiber and cause ablation or melting of the end facet. To minimize this effect, we chose the focal length of the coupling lens to produce a laser focal spot that is only slightly smaller than the core diameter of the crystal fiber. Backward propagating Raman light is split into Stokes and anti-Stokes paths. Additional bandpass filters of roughly 30 nm bandwidth are used in both paths to remove unwanted blackbody radiation or stray light. These filters permit collection of most of the Raman lines in either sapphire or YAG fibers [30]. The Raman pulses are focused onto avalanche photodiodes, which are sampled at about 2 GHz. A square-wave generator with an adjustable frequency ranging from about 5–20 kHz is used to time laser pulses and subsequent digital acquisition.

Careful consideration must be given to the design of fiber optic probes for energy system measurements. In general, energy systems are very large and require significant standoff distances between interrogation equipment and harsh-environment measurement areas of interest. For this reason, our probe systems often employ a standard fused silica multimode fiber as a patch cable between the interrogator and the single-crystal optical fiber probe. Such standoff cables can range from 1–50 m in length, while measurement probes generally range from 1–10 m in length. For maximum optical transmission coupling efficiency, a fusion splice is often used to couple the fused silica lead-in fiber to the single-crystal fiber probe [31,32].

**Figure 6:**
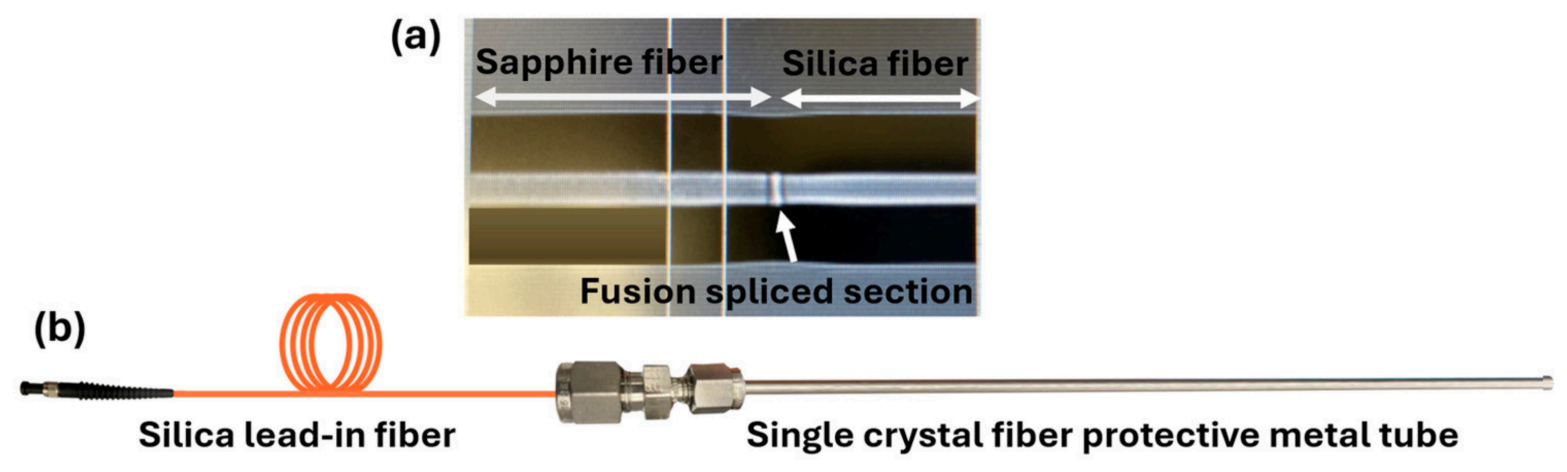
Fabrication of a Raman-DTS probe. (**a**) Close-up image of a 125 um-diameter Sapphire fiber fusion-spliced with a silica multimode fiber (MMF). (**b**) Packaged sensor probe comprising a silica MMF lead-in fiber and a single-crystal sapphire fiber sensor housed within a protective metal tube.

A fusion splice of this type as shown in Figure 6a is produced using a large diameter fiber fusion splicer (S178 LDF, OFS Fitel, Norcross, GA, USA) with a custom splicing program. The splice arc does not melt the single-crystal fiber; instead, it heats the crystal fiber to permit wetting while the multimode lead-in fiber is fully melted and adhered to the crystal. Standard polyvinyl-chloride or armored stainless steel protection tubing is used to protect the multimode lead-in fiber from abrasion or damage in a power-plant environment, depending on the specific installation area requirements. The fusion splice and crystal fiber probe are encapsulated in one or more outer sleeve materials depending on the application of interest. In the simplest configuration, a stainless steel or nickel alloy tube is used to encapsulate the crystal fiber probe as shown in Figure 6b. For higher temperature applications, probes can be made of ceramic or polycrystalline sapphire tubing similar to a standard thermocouple protection tube. Multilayer designs with internal ceramics, MgO powder, and external sheaths are other packaging options for mechanical robustness. In a permanent installation, a Raman-DTS system could be installed along with a specialty multimode visible wavelength fiber optic switch to allow operation with multiple fiber optic probes using a single interrogator.

## 5. Distributed Temperature Measurements

The Raman-DTS system for single-crystal optical fiber described here was designed to have measurement capabilities that are largely compatible with a wide variety of energy generation systems and components. We report a spatial measurement resolution of roughly 2–5 cm, depending on the exact type, diameter, and length of the fiber probe being implemented as well as the measurement rate [6]. Larger core diameter and larger propagating NA yields coarser spatial resolution. The resolution was verified using fiber translation tests, in which the fiber was translated in 1-cm increments through a previously characterized hot zone. The specific spatial resolution for the fiber described in these experiments was about 4 cm. Temperature resolution is about 1 °C, with absolute temperature accuracy (error versus absolute verified measurement) ranging from 5–30 °C depending on many of the same factors. We calculate a greater error contribution from stary collected blackbody radiation at the Stokes wavelength than from uncertainty propagation or from other sources. Temperature accuracy was verified using a thermo-well calibrated thermocouple that exhibited a +/−3 C accuracy itself. The maximum measurement errors reported were somewhat larger than those achieved by fiber Bragg Gratings in sapphire, although we note that fully distributed techniques do not require grating inscription [33]. The system can measure at a rate of 1 Hz, but most applications generally require far less speed due to the large thermal mass involved in large-scale systems. Tradeoffs in operating characteristics are indeed possible via specific operating parameters and settings. For example, increases in absolute accuracy can be obtained with lower sampling rates. Coarser spatial resolution can allow higher measurement rates. We also note that pulse spread plays an important role in spatial resolution as the length of the multimode crystal fiber increases. For a 110 µm core multimode fiber with a NA of about 0.22, mode coupling accounts for about 50 ps of pulse spread after about a meter in fiber length. This increases to about 200 ps after 2 m and stabilizes to more than 5 ns for 10-m lengths due to full relaxation into all available optical modes. This means that spatial resolution is somewhat worse as fibers get longer. Where modal dispersion pulse broadening scales linearly with length as:

$$\Delta \tau =\frac{L*{NA}^{2}}{2*c*n}$$

in a step index multimode fiber. For comparison, a single mode fiber with 350 ps pulses would exhibit an approximately 3 cm spatial resolution which does not change appreciably along the length. Our 0.22 NA multimode system exhibits about 4 cm at 1 m length, with resolution decreasing towards 5 cm at 2 m sensing length. For a highly multimode unclad 110 µm core sapphire fiber, lengths of tens of meters are not very useful, because modal dispersion would deteriorate spatial resolution to tens of centimeters or more than half a meter at those lengths. This highlights the importance of reducing modal volumes using doping or other methods described here for long-length single-crystal optical fiber probes.

**Figure 7:**
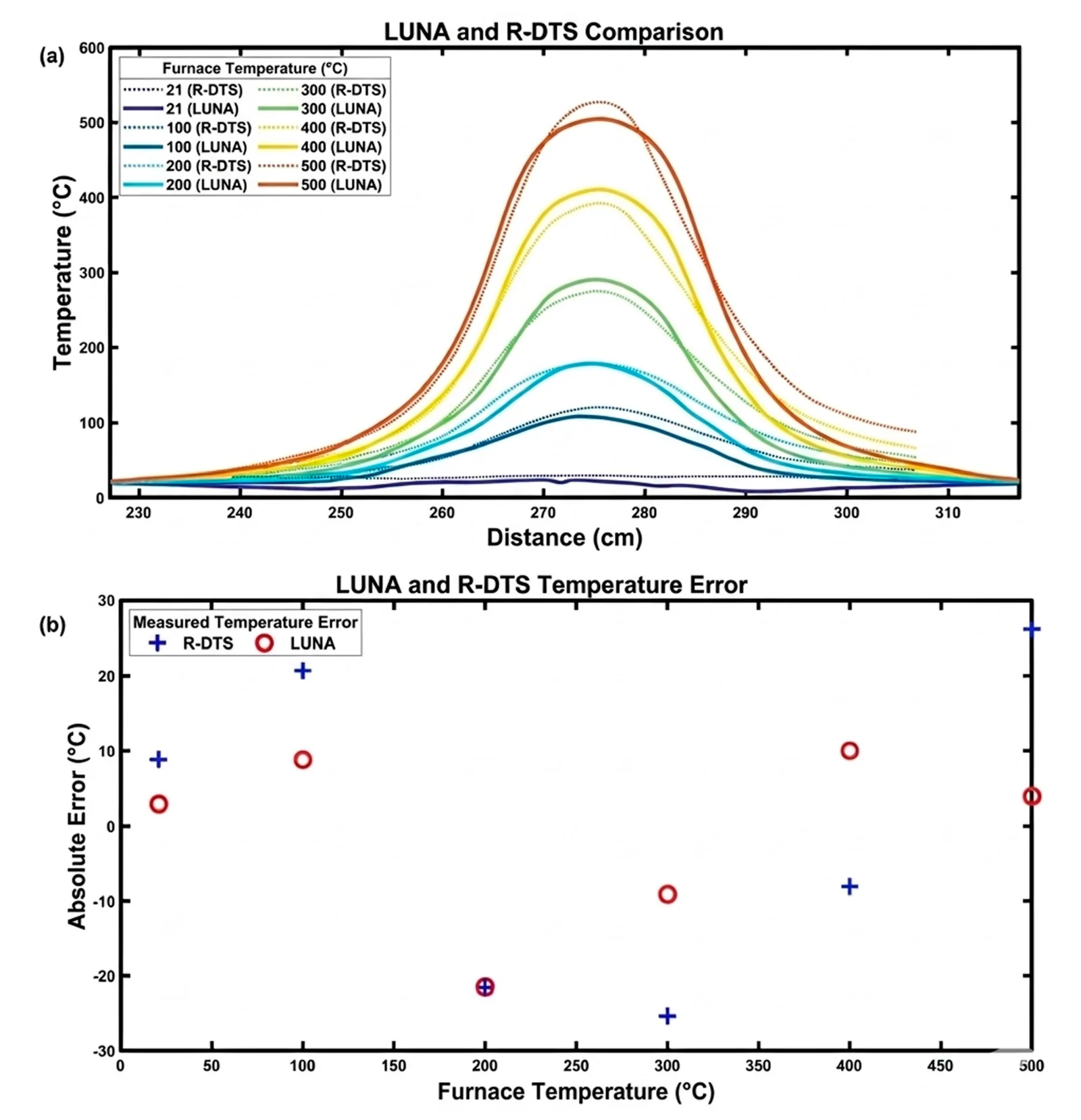
Distributed temperature measurements. (**a**) Temperature profiles calculated via measurements taken with the LUNA OFDR system and our custom-built Raman-DTS system, (**b**) Error in temperatures measured by two DTS systems. Note, both systems performed comparably on temperature accuracy.

To provide a clear comparison of capabilities with commercially available fibers, we installed a standard air-clad sapphire optical fiber approximately 1-m in length in a tube furnace alongside a single-mode gold-coated optical fiber (Fibercore Ltd. (Southampton, UK). The single mode fiber was connected to a commercial interrogation system (LUNA Odisi 6000, Luna Innovations Inc., Blacksburg, VI, USA). This unit utilizes an OFDR method that operates via Rayleigh backscattering, as opposed to Raman. It exhibits a 100 m maximum sensing range, a 0.65 mm maximum spatial resolution, and includes a temperature measurement module. This type of OFDR sensor generally cannot distinguish strain and temperature directly, without the use of a de-coupled fiber to distinguish the two parameters. Raman OTDR in contrast is not strain sensitive. Although this sensor system is generally utilized with LUNA sensor fibers for temperature measurement up to 300 °C, we utilized the gold-coated fiber to access temperatures up to about 500 °C. Accuracy with the lower temperature LUNA sensor fibers is purported to be below about 3 °C, although accuracy is not published for higher temperature fibers such as the specialty gold-coated fiber used for this effort. Beyond about 700 °C, the gold-coating will spall off the silica fiber, and the sensor will cease to operate. Given these constraints, we performed tube-furnace (GSL−1800X, MTI corp., Richmond, CA, USA, 20 cm heated zone, 0.7 m tube length) testing up to about 500 °C to compare the accuracy and capabilities of both sensor systems for the first time. These tests were designed to provide some details regarding the capabilities of our Raman system using a fiber that is commercially available to highlight what can be accomplished even in the absence of the availability of clad fibers like those described here (which are not commercially available), and not to establish superiority or practical competitiveness, as such comparisons would likely be dependent on use case, cost, and other factors. Testing of this sensor probe was performed over an extended period of time [6] with numerous ramp and hold cycles over a period of at least 3 weeks in the hot zone to verify robustness of measurements in this range. In prior work, higher temperatures have been explored up to at least 1400 °C [6]. Figure 7 shows distributed measurements using both the Raman-DTS and the LUNA-DTS systems.

The furnace was ramped from room temperature to 500 °C and was allowed to stabilize at each temperature for 15 min before a measurement was taken with both distributed sensor systems. Both instruments provide reasonable measurements of the shape and magnitude of the furnace hot zone over ~1 m test fiber length. In the LUNA OFDR system, it was necessary to find curve fitting parameters to translate the measured spectral frequency shift into temperature. This is thought to introduce most of the measurement errors associated with that system while using a large range of applied temperatures. In the Raman system, most of the measurement error (especially on the far end of the sensor) is believed to be due to thermally induced transmission changes in the single-crystal material which are not necessarily exactly equal for Stokes and anti-Stokes wavelengths. Regardless, both systems could produce similar hot-zone measurements of the target system. Most of the differences in curve-shape are largely due to the spatial resolution evident in either system.

## 6. Conclusions

Single-crystal optical fibers present a compelling new path for conducting fully distributed sensing in extremely harsh environments of energy generation systems. Although unclad versions of these types of fiber have been available for decades, emerging new methods for fiber cladding are re-invigorating interest in their use for laser and sensing applications. Effective cladding methods will reduce modal volumes, enable high-resolution sensing at moderate lengths, and provide protection from contamination and degradation in challenging environments. As these fibers are expected to remain few-modes or multimode over many relevant wavelength ranges, time-domain interrogation approaches, particularly Raman-based OTDR methods, remain well suited for distributed temperature measurements in these materials. Although the demonstrations and perspectives described here are closer to laboratory-scale than full industrial deployment, we plan to embark on a series of extensive field-testing campaigns and collaboration with industrial partners to bring the technology closer to full deployment and commercialization. As advanced coal, oil and gas, and nuclear systems continue to operate at higher temperatures and in more harsh environments, single-crystal fiber sensors are likely to play an increasingly important role in regions where silica-based sensors cannot provide adequate thermal or chemical robustness. Continued improvements in crystal growth, core-cladding engineering, and a reduction in both both optical loss and number of propagating optical modes will be essential to extending sensing length, improving spatial resolution, and achieving reliable high-temperature distributed sensing capabilities in next-generation energy systems.

## Data Availability

The raw data supporting the conclusions of this article will be made available by the authors upon reasonable request.

## Abbreviations

The following abbreviations are used in this manuscript:

CO_2_	Carbon dioxide
DTS	Distributed temperature sensing
FUT	Fiber under test
GHz	Gigahertz
kHz	Kilohertz
kW	Kilowatt
LHPG	Laser heated pedestal growth
MMF	Multimode fiber
NA	Numerical aperture
OFDR	Optical frequency-domain reflectometry
OTDR	Optical time-domain reflectometry
PID	Proportional-integral-derivative
Raman-DTS	Raman distributed temperature sensing
RI	Refractive index
YAG	Yttrium aluminum garnet
ZnSe	Zinc selenide
